# Personal genome testing: Test characteristics to clarify the discourse on ethical, legal and societal issues

**DOI:** 10.1186/1472-6939-12-11

**Published:** 2011-06-14

**Authors:** Eline M Bunnik, Maartje HN Schermer, A Cecile JW Janssens

**Affiliations:** 1 Dept. of Medical Ethics and Philosophy of Medicine, Erasmus University Medical Center, Office AE-340, PO Box 2040, 3000 CA Rotterdam, The Netherlands; 2 Dept. of Epidemiology, Erasmus University Medical Center, Office EE-21-40b, PO Box 2040, 3000 CA Rotterdam, The Netherlands

## Abstract

**Background:**

As genetics technology proceeds, practices of genetic testing have become more heterogeneous: many different types of tests are finding their way to the public in different settings and for a variety of purposes. This diversification is relevant to the discourse on ethical, legal and societal issues (ELSI) surrounding genetic testing, which must evolve to encompass these differences. One important development is the rise of personal genome testing on the basis of genetic profiling: the testing of multiple genetic variants simultaneously for the prediction of common multifactorial diseases. Currently, an increasing number of companies are offering personal genome tests directly to consumers and are spurring ELSI-discussions, which stand in need of clarification. This paper presents a systematic approach to the ELSI-evaluation of personal genome testing for multifactorial diseases along the lines of its test characteristics.

**Discussion:**

This paper addresses four test characteristics of personal genome testing: its being a non-targeted type of testing, its high analytical validity, low clinical validity and problematic clinical utility. These characteristics raise their own specific ELSI, for example: non-targeted genetic profiling poses serious problems for information provision and informed consent. Questions about the quantity and quality of the necessary information, as well as about moral responsibilities with regard to the provision of information are therefore becoming central themes within ELSI-discussions of personal genome testing. Further, the current low level of clinical validity of genetic profiles raises questions concerning societal risks and regulatory requirements, whereas simultaneously it causes traditional ELSI-issues of clinical genetics, such as psychological and health risks, discrimination, and stigmatization, to lose part of their relevance. Also, classic notions of clinical utility are challenged by the newer notion of 'personal utility.'

**Summary:**

Consideration of test characteristics is essential to any valuable discourse on the ELSI of personal genome testing for multifactorial diseases. Four key characteristics of the test - targeted/non-targeted testing, analytical validity, clinical validity and clinical utility - together determine the applicability and the relevance of ELSI to specific tests. The paper identifies and discusses four areas of interest for the ELSI-debate on personal genome testing: informational problems, risks, regulatory issues, and the notion of personal utility.

## Background

In discussions on ethical, legal and societal issues (ELSI) surrounding genetic testing, there is no longer any single satisfying definition of what constitutes 'a genetic test'. Practices of genetic testing are becoming more and more heterogeneous, not only with regard to the setting and purpose of testing, but also with regard to the technical aspects of the tests themselves. Some of these technical differences between genetic tests are ethically significant or have implications for legal or societal issues. Therefore, a clear understanding of the relevant test characteristics of genetic tests is a necessity for any meaningful discussion of the ELSI surrounding genetic testing.

Over the last decades, new technologies for genetic testing have been developed that differ in many respects from those used in traditional clinical genetic testing for monogenic diseases. One important development is the advent of personal genome testing on the basis of genetic profiling for the prediction of common multifactorial diseases. Multifactorial diseases, such as cardiovascular diseases [1], age-related macular degeneration [2], type 2 diabetes [3], clinical depression [4], and many types of cancer [5], are caused by intricate interplays of multiple genetic factors and non-genetic factors. Through an analysis of those genetic factors, an individual's genetic susceptibility to multifactorial diseases can be determined. Personal genome testing companies are currently offering such risk prediction services directly-to-consumer, thereby raising a range of new ELSI.

With this paper, we aim to clarify the relations between the more technical characteristics of a genetic test and the ELSI with which the test is associated. We believe that a thorough understanding of the technical characteristics of personal genome tests themselves forms a necessary basis for all further ELSI-discussions in the field. Our focus on the test characteristics implies that, in this paper, we will not be able to discuss other aspects that are relevant to ELSI-discussions, such as characteristics of the diseases tested for, or the settings in which tests are offered. Although there are moral differences, for example, between the offering of personal genome tests by private companies and the offering of the same tests by public health care systems, or between testing for diseases for which there are treatment options available and testing for diseases for which there are no such options, these differences are not the main subject of this paper. As personal genome tests are currently offered almost exclusively in a direct-to-consumer context, we take that context as the background to our discussion.

First, we will introduce the practice of personal genome testing. In the second section, we will distinguish and briefly discuss the following four key test characteristics of genetic testing: from targeted to non-targeted testing, analytical validity, clinical validity and clinical utility. The third section of the paper discloses and discusses four major areas of implications of these test characteristics for the ELSI-debate.

## Discussion

### I Personal genome testing

Personal genome testing for multifactorial diseases is conducted on the basis of genetic profiling. In a genetic profile, multiple genetic variants are combined that are associated with increased or decreased risks for a particular multifactorial disease. Presently, single nucleotide polymorphisms (SNPs) are used within genetic profiles [6]. SNPs are variations of a single nucleotide, the smallest building block of DNA. Most common SNPs that are known today convey only minor risks [7]. They are distinguished from mutations that cause monogenic diseases, which are rare but convey large risks.

For almost a decade, companies have been offering genetic profiles based on SNPs directly to consumers via the Internet. Initially, personal genome testing companies marketed single profiles for specific health conditions, or a limited set of profiles for related diseases [8]. Today, companies are offering genome-wide profiling services that yield a multitude of profiles not only for common multifactorial diseases, but also for non-medical traits [9]. In recent years, personal genome testing companies have been at the centre of an ongoing critical debate on their ethical, legal and societal issues (ELSI) [10]. Within the ELSI-debate, personal genome testing services have been criticized for their lack of clinical validity [11-13], for being premature [14] or a waste of private and public money [15,16].

Other direct-to-consumer companies are starting to offer genetic profiling on the basis of whole-genome sequencing technology: the analysis of all three billion base pairs.^1 ^Whether providers make use of genome-wide SNP-analysis or whole-genome or exome sequencing technology, however, the prediction of common multifactorial diseases and other complex traits will continue to be based upon multiple genetic variants, and thereby upon the construction of genetic profiles. In this respect, therefore, the scope of this paper is wide and encompasses all potential forms of personal genome testing based on genetic profiling: current and future, commercial and clinical forms, including sequencing technologies.

### II Test characteristics

There are four key test characteristics relevant to the ELSI-debate to be discussed: from targeted to non-targeted testing, analytical validity, clinical validity and clinical utility (see Table 1).

**Table 1:** Test characteristics of personal genome testing and their implications for the discourse on ELSI

Test characteristic	Implications	ELSI
		
From targeted to non-targeted testing	Quantity and complexity of information	The information problem - Informed consent - Information provision (pre-test and post-test) - Informational updates - Incidental findings

Analytical validity	High analytical validity	Regulatory issues

Clinical validity	Generally poor clinical validity (validity varies per disease tested for)	- Psychological risks, health risks and societal risks - Regulatory issues

Clinical utility	Generally poor clinical utility (utility varies per disease tested for)	- Personal perspectives on utility - Changing interpretations (fluidity of information)

### 1. From targeted testing to non-targeted testing

In targeted testing, the patient or consumer is tested for a single particular disease. Clinical genetic testing is by definition targeted, because clinical geneticists are scrutinizing the genome for risks of a particular monogenic disease, or, in the process of diagnosis, for one particular genetic disease to explain clinical symptoms. There are targeted forms of genetic profiling, where an individual's genetic susceptibility to a particular multifactorial disease is estimated on the basis of a set of genetic variants across the genome [8]. Personal genome testing companies have been marketing multi-targeted testing for a limited range of diseases [9]. Most present-day personal genome testing companies, however, offer *non-targeted *forms of genetic profiling: they genotype millions of SNPs and construct profiles that convey personal risks for a large and continuously increasing number of multifactorial diseases and other genetic traits.^2^

#### 1.1 Quantity and complexity of information

Non-targeted forms of personal genome testing offer unequalled quantities of information on the basis of one single laboratory assay. There are companies that offer predictive information about dozens of multifactorial diseases and other phenotypic traits simultaneously.^3 ^These quantities of information may be too large for patients or consumers to process. The sheer amount of information conveyed by personal genome testing poses problems of information overload as well as feasibility issues with regard to informed consent requirements.

It is well-known from clinical genetic testing that genetic information is generally found to be complex. This is even more true in the context of multifactorial diseases, for not only are multiple genetic variants, each with their own effect sizes, involved in the causation of multifactorial diseases, there are also environmental factors at play. Multifactorial disease risks are probabilities: they are relative risks and may diverge only slightly from average population risks. Empirical studies have shown that many people find probabilistic information difficult to interpret [17]. People are inclined to perceive risks that are only slightly increased or decreased nonetheless in a dichotomous manner, as either 'high' risk or 'low' risk [18]. They have little prior knowledge of the genetics of multifactorial diseases [19], and feel incapable of understanding complex genetic risk information [20]. In personal genome testing, for example, consumers may not always understand that negative test results or lower-than-average risks are no guarantee for remaining healthy. In non-targeted forms of testing, the problems of complex information are exacerbated by the enormous quantity of information.

#### 1.2 Incidental findings

An implication of the current shift from targeted to non-targeted testing, is that non-targeted testing affects the ELSI-issue of incidental findings. Incidental findings are test outcomes that are unintended or unexpected, for example: SNP-data which are not yet of predictive ability, but may become so in the future as new SNP-disease associations are being discovered. In non-targeted testing, the potential for this type of incidental test outcomes is much greater than in targeted testing, simply because it yields a much larger data set, the significance of which is not yet fully understood. Consequently, ethical issues that have traditionally been associated with clinical genetic testing, such as problems with the disclosure of incidental or future findings and concurrent psychological risks, may at first glance become more urgent for non-targeted personal genome testing. The issue will be further discussed in section III,1,4.

### 2. Analytical validity

The analytical validity of a genetic test refers to the accuracy with which the laboratory assay measures the genetic variant it is designed to identify. This notion includes the capacity that the test will be positive if the genetic variant is present (analytic sensitivity), and negative if it is absent (analytic specificity) [21]. In the ethical evaluation of clinical applications of genetic testing, the analytical validity has traditionally been a primary criterion [22]. It is derived from basic consumer rights: a genetic test, like any other product, ought to 'conform to contract' and be as described on its labeling.

Some of the early genetic profiling companies were selling nutritional supplements based on targeted genetic profiling tests of unproven analytical validity [23,24]. There has been a sharp critical debate [25,26], and in many countries, regulatory bodies have become more alert on direct-to-consumer genetic testing services [27,28]. Presently, however, partly as a result of more responsible conduct of business, analytical validity is no longer a major topic in the ethical debate on personal genome testing. The new generation of personal genome testing companies is analytically reliable,^4 ^such that most current "genomic assays have high sensitivity and specificity for measured genetic variants" [29]. The industry strives for transparency and truth-in-advertising, and discloses detailed information on the technologies used for their laboratory assays.^5 ^Although the policy-making process is still ongoing, many companies have responded with improved analytical validity.

### 3. Clinical validity

Whereas analytical validity refers to the quality of the laboratory assay, clinical validity is a criterion of the *interpretation *of assay results, a criterion of the *test *[30]. Clinical validity is measured by the predictive ability or discriminative power of the genetic variant: its ability to classify individuals as those who will develop the disease and those who will not [21]. Since the effects of SNPs on disease risks are so small, most current personal genome tests lack that discriminative power. By far the largest proportion of patients or consumers will demonstrate personal risks for multifactorial diseases that approximate the average population risk: these risks will prove to be only slightly lower or slightly higher [31]. A genetic profile that yields individual outcomes between 14% and 21% for major depressive disorder cannot be clinically meaningful when the average population risk is 17%. There will only be few consumers with absolute risks that diverge sufficiently from the average population risk to be clinically significant. Therefore, in contrast to that of clinical genetic testing for monogenic diseases, the clinical validity of genetic profiling for multifactorial diseases for the purposes of individual disease risk prediction, so far, has remained rather limited.

Statistical studies are finding that the addition of relatively significant SNPs to conventional risk models does not always improve their discriminatory power, for example: genetic information has not been capable of improving traditional prediction models for type 2 diabetes based on phenotypic risk factors and family history [32].^6 ^Genetic profiles are expected to gain some clinical validity in the future as they are refined and expanded to include more SNPs or other genetic variants, especially as they may become based on sequencing technologies [33]. Further, with the inclusion of environmental factors into risk profiles for multifactorial diseases, their clinical validity may gradually increase even further.

### 4. Clinical utility

In recent years, there have been conceptual discussions of the criterion of clinical utility, which has been widely used for the (ethical) evaluation of genetic screening programmes [34]. Roughly, there are three perspectives on utility: the public health perspective, the clinical perspective and, finally, the personal perspective [35], which will be discussed in the next section. Within the public health perspective, in order to have utility, a genetic test must improve health outcomes in terms of morbidity or mortality on the societal level, be cost-effective, and produce benefits that outweigh the risks [36]. The principle of clinical utility requires test results to provide patients with 'actionable options' for prevention or treatment that are accessible and safe and that have been proven to be effective. From the clinical perspective, genetic information must alter clinical management, influence therapeutic decision-making, or lead to better prediction models [37]. Current personal genome testing for multifactorial diseases is not likely to pass the test in either perspective.

Within a clinical perspective, genetic profiling for, for example, type 2 diabetes may become clinically valid in the future, and thus capable of disclosing informative and reliable risks, but it may not necessarily become clinically *useful*. For it is not always clear *what to do *with a slightly increased personal risk of developing type 2 diabetes, or whether, say, a 28% absolute chance is a sufficient reason to take preventative action or to alter clinical management. Within a public health perspective, there are established preventive measures available for type 2 diabetes, such as weight loss, exercise, and smoking cessation. These measures are inexpensive, harmless and, in fact, beneficial to the whole of the population. Since it would be worthwhile to prescribe these measures to both high-risk and low-risk individuals for type 2 diabetes, however, the usefulness of the genetic test is minimal. As long as personal genome tests continue to be of minimal clinical utility, they will not find their way into the clinic.

#### 4.1 A personal perspective on utility

The third and personal perspective on clinical utility takes a broader and more subjective view, for it is defined by patients or consumers themselves. It allows for non-medical, particularly psychological motivations for genetic testing, such as solace [38], family planning or preparation for the future [29]. In clinical genetics, non-medical motivations are often part of the counseling and decision-making processes, paradigmatically in genetic testing for Huntington's disease, for which there are no preventative or therapy options available. Such testing has 'clinical utility' from a personal point of view: test outcomes may offer either reassurance or certainty, and, subsequently, the psychological benefits of 'knowing' and the ability to make important life decisions, including, importantly, reproductive decisions. In the context of new technologies for genetic profiling, critics have proposed to broaden the concept of 'personal utility' much further, so as to include the value of 'information per se' [35], the desire to be reassured, and something like the fun aspect or the entertainment value of knowing about one's genes.

#### 4.2 Changing interpretations

The clinical utility of genetic profiles is affected by a further test characteristic, namely that of changing assay interpretations. As genetics research proceeds, more and more gene-disease associations are discovered. Newly found genetic variants are included in ever more extended genetic profiles. As personal genome testing companies offer updates of their profiles, however, the companies' test outcomes are therewith subject to change over time [39]. On the basis of the same biological sample and the same laboratory assay, companies may present diverging, and even contradictory, test outcomes over time. A consumer reports:

"When I had my genome scanned a year and a half ago, using deCODEme's direct-to-consumer genotyping service, the results suggested my lifetime risk of having a heart attack was slightly higher than usual, at 1.12 times the average. When I logged on to my profile again today, though, I discovered that my chances of developing the same condition now appear to have shot up: my relative risk is now 1.28, giving me a 62.7 lifetime risk of having a heart attack. [....] What has changed, however, is the data that the company uses to calculate genetic risk. In May, deCODEme added six new genetic variations to its algorithm for assessing its customers' risk of having a heart attack, on the back of new research [40]."

The probability of receiving contradictory results over time is quite high. A modeling study on genetic profiling for type 2 diabetes has shown that the update from one relatively strongly predictive SNP by an additional 17 less predictive SNPs, causes 34% of the study's population to switch risk categories either from above average risk to below average risk or vice versa [41]. Due to changing interpretations, personal genome tests yield fluid test results.^7^

### III. Implications for the ELSI debate

In this section, the implications of the test characteristics of personal genome tests for the discourse on ELSI will be discussed. As shown in section II, 3, the test characteristic of analytical validity is no longer a topic of major concern in the ELSI-debate since the advent of a new generation of personal genome testing services. Therefore, it will not be discussed any further. The three remaining test characteristics (together) do lead to ELSI that are of importance to the current debate on personal genome testing in a direct-to-consumer context: informational problems, risks, regulatory issues and notions of utility.

### 1. Non-targeted testing: The information problem

The most important ELSI-issues in personal genome testing are related to information. Within the ELSI-debate, it has already been argued that priority ought to be given to informational problems; critics have stated, for instance, that both public and professional institutions ought to take up the responsibility to inform the general public, to raise awareness of the risks of direct-to-consumer genetic testing [42], and to develop reliable information sources for consumers as well as physicians [43]. The previous section has brought to light a subset of test characteristics that together lead to the problem of information within *non-targeted *genetic profiling: quantity, complexity, and fluidity of information. The informational problem is associated with the practice of non-targeted genetic profiling itself, whether within or outside the clinic, now or in the future.^8^

Discussions of the difficulties surrounding the provision of genetic information are not new: in clinical genetic testing, patients are routinely offered extensive genetic counseling prior to consenting to undergo genetic testing. During counseling sessions, the patient receives detailed information about the disease, the genetic component thereof, the testing procedure, possible outcomes, therapeutic options, implications for reproductive choices and possibilities, consequences for the family, the communication of possible risks to relatives, social implications, privacy issues, potential adverse effects on employment and insurance, etc. [44]. Ideally, a well-considered decision is made by the patient and informed consent is obtained on the basis of accurate and detailed information.

In targeted genetic profiling, it will not necessarily be difficult to meet these widely endorsed high standards for informed consent: in all likelihood, the genetic counselor will be able to deal with most relevant aspects of genetic testing for any single multifactorial disease within the scope of a few counseling sessions. Non-targeted genetic profiling, however, poses the problem of *exceptional quantities *of information on dozens to over a hundred different diseases. It will be very arduous, if not impossible, to inform patients or consumers beforehand in detail on all relevant aspects for so many diseases without inducing information overload and therewith foregoing the actual aim of informed consent [45].

The information problem will be even greater for whole-genome sequencing, which will reveal not only SNPs that are weakly associated with risks of multifactorial diseases, but also highly predictive mutations that cause monogenic diseases. As a consequence, the direct-to-consumer availability of whole-genome sequencing services might imply serious psychological risks as well as health risks, and thus will have important ELSI-implications of its own. These issues, albeit pressing, are beyond the scope of this paper, which focuses specifically on personal genome testing in the context of *multifactorial *diseases.

#### 1.1 Informed consent

Informed consent is intended to protect individuals against unwanted procedures and to acknowledge the individual's capability to decide for himself or herself whether or not to receive information with regard to their health status or to undergo a physical examination or intervention. Informed consent "allows individuals to exercise their fundamental right to decide whether and how their body, body parts and associated data will be used" [46]. The right to respect for autonomous decision-making and the protection against misuse of human bodies are among the pillars of health care ethics [47] which hardly any of us will desire to give up. However, the feasibility of informed consent requirements is seriously threatened by the informational problems associated with personal genome testing.

There are three basic responses to the problem of informed consent: first, it could be argued that, if fully specific informed consent is not possible for non-targeted genetic profiling, these services ought not to be made available, at least not in any non-supervised, direct-to-consumer fashion [13]. Second, if it is acknowledged that full and accurate information is not always possible or even available in the genetics of multifactorial diseases, it could be concluded that the ideal of informed consent has become outdated and (for that domain of genetics) had best be abandoned altogether. Third, if it is accepted that the provision of information will necessarily be incomplete, it could be claimed that the procedure ought to focus on the information that is *most necessary and indispensable *for consumers to give valid consent and to effectively prepare themselves for personal genome testing. Versions of the third ethical position have already been proposed [48-50]: they are to serve the value of consumer autonomy, for they preserve access to personal genome testing and allow for liberty of choice. At the same time, they recognize that patients have a need for and a right to information - for without adequate information, freedom of choice is meaningless.

We also find the third position more convincing than the other two, and believe that informed consent is both possible and required for direct-to-consumer personal genome testing. Further discussion and research are needed to determine exactly what (selection of) information (and to what level of detail) is most crucial for valid informed consent. For example, patients or consumers may need to be informed beforehand in general terms about changing interpretations as a consequence of ongoing genetics research. Also, they may need to be made aware of ELSI-related differences between diseases or types of diseases. Finally, for instance, patients or consumers may need to be given the opportunity to decide in advance what kinds of genetic information they do and do not wish to receive as part of an informed consent process.

#### 1.2 Information provision: pre-test and post-test

In personal genome testing for multifactorial diseases, consumers or patients are confronted with a double uncertainty: genetic risks in themselves are probabilistic, and the clinical validity and utility of these risks are doubtful. Patients or consumers are likely to experience difficulties not only during the process of informed consent, but also afterwards, when they receive and interpret their test outcomes. In recent years, there has been disagreement over the way in which test results ought to be provided in personal genome testing, particularly over the question whether face-to-face discussions with a genetic counselor are deemed necessary. Some have stated that all genetic testing ought to be accompanied by genetic counseling [51-54], in order to warrant accurate interpretations of test results.^9 ^Live discussions with genetic counselors are required in complex cases, such as incidental findings. Others however have argued that "in the context of possible widespread introduction of genetic screening for common diseases, genetic counseling should be concentrated on those conditions that threaten life or have a serious impact on the ability to live life fully" [55]. From the second position it follows that the need for face-to-face counseling does not apply to present-day personal genome testing for multifactorial diseases, for serious psychological impact is not to be expected (see section III, 2). This more liberal position, which we believe is preferable to the more stringent position, would allow providers of personal genome testing services to suffice with the provision of adequate written information, both pre-test and post-test.

#### 1.3 Information updates

Most companies or institutions tend to retain biological samples or genetic data sets from their clients or patients [56]. In the future, as new discoveries will occur within the field of human genetics, new and important disease risk information could potentially be deduced from the original data. It is still a matter for debate whether companies or institutions have a moral or legal duty to gather that information and to re-contact their clients or patients. Roughly, there are three possible stances: firstly, companies or physicians do have such moral duty and ought, for instance, to provide regular updates on the clinical interpretation of purchased genetic data sets. Secondly, consumers or patients may prefer to decide individually whether or not they wish to be contacted in the future, and on what conditions. They could be given the opportunity beforehand to express their wishes with regard to future findings. Thirdly, it is a moral responsibility of patients themselves to become or to remain informed on scientific proceedings or to re-contact their companies or physicians if they wish to obtain updated information. The distribution of moral responsibilities, we believe, may depend largely on contextual variables, discussion of which, however, is beyond the scope of this article. On a general level, we think that there are differences in the extent of moral responsibility between companies on the one hand and physicians or health care institutions on the other hand, since the latter can be said to have a stronger professional duty than the former to provide their patients with medical care and follow-up.

#### 1.4 Incidental findings

In section II, 2, the increased potential for incidental findings has been mentioned for non-targeted personal genome testing. However, it could contrarily be argued that, in non-targeted testing, no finding is incidental. The aim of non-targeted testing is to convey *a lot of information *on the basis of one biological sample. Personal genome tests are marketed and presented to the public to include a wide variety of SNP-trait and SNP-disease associations, and companies tend to update risks and include more diseases as soon as new SNP-disease associations have been validated.^10 ^If the aim is to look for everything, the notion of an incidental finding loses its original meaning. Keeping the patient or consumer perspective in mind, however, it is important to note that they may not always be prepared for finding everything: they may still be surprised by some of the (for them) incidental findings. It could be argued that they ought to be made aware of a right not to know certain (types of) information, as part of an informed consent process.

### 2. Clinical validity and utility: Psychological risks, health risks and societal risks

Theoretically, there are three types of risks to be expected from personal genome testing, as a result of its limited clinical validity: psychological risks, health risks, and societal risks. First, the complexity of genetic information together with the limited predictive ability of the tests themselves, render personal genome testing susceptible to misunderstanding and misinterpretation. Consumers could feel worried about overestimated disease risks and could suffer from undue anxiety. Critics have worried that a class of 'worried well' might come into being [57-59], especially since some present-day personal genome testing companies tend to exaggerate the clinical validity of their services.

On the other hand, we believe that adverse psychological effects that are well-known from clinical genetic testing, such as emotional distress, depression or survivor guilt as a result of test outcomes, are *not *to be expected from genetic profiling to the same extent [60]. As test outcomes for multifactorial diseases lack clinical validity, they are much more likely to lead to epistemic uncertainty than to the major psychological impact known from clinical genetics. Moreover, empirical research has shown that even genetic testing of high clinical validity, such as for hereditary breast or colorectal cancer syndromes, leads to much less psychological harm than traditionally thought [61]. The provision of genetic information of more limited clinical validity, such as in type 2 diabetes, appears not to adversely affect individuals at all [62,63]. Thus, the psychological risks involved in personal genome testing are likely to be overestimated. At the same time, however, we acknowledge that the potential for misinterpretation and misunderstanding of complex genetic information cannot be stressed too frequently.

Secondly, it is frequently argued that there are health risks implied in personal genome testing for multifactorial diseases [12][25]. False reassurance on the basis of testing of limited clinical validity is thought to lead patients or consumers to adopt unhealthier lifestyles, to omit standard preventative measures, or to forego regular screening, thus causing harm to their health. The following example constitutes a worst-case scenario: there are companies that analyze sets of SNPs for calculating the risk of colorectal cancer.^11 ^These SNPs are associated with common, non-hereditary forms of colorectal cancer and are very weak risk factors of limited clinical validity. The genetic profile offered does not include highly penetrant mutations involved in the causation of 5-10% of cases of monogenic hereditary colorectal cancer syndromes [64]. Hypothetically, consumers from high-risk families could feel reassured on the basis of a few negative SNPs of limited clinical validity, whereas their genomes have not been analyzed for other, higher-risk mutations. In reality, however, we expect that at-risk consumers are likely to present themselves at clinical genetics centers for testing, so these cases will be rare in a direct-to-consumer context. At present, there is no empirical evidence to back up the fear of false reassurance. There are indications that the impact upon lifestyle is minimal in most consumers of personal genome testing [63]. Thus, we believe that fears of health risks may be overstated.

Finally, there are at least two perceived societal risks involved in personal genome testing of low clinical validity: indirect economic risks, and loss of public confidence in genetics research and applications. Firstly, on the basis of personal genome test results, consumers may turn to their physicians for advice, follow-up research or medication. As the clinical validity of test results are uncertain, most of the follow-up will be unnecessary while it does drive up the costs of public health care [15][65]. Empirical studies suggest that consumers are indeed likely to consult their physicians for help with the interpretation of tests results obtained from personal genome testing companies [16][20]. Thus, private spending on direct-to-consumer personal genome testing may ultimately lead to higher collective costs of public health care. Secondly, it has been pointed out that the commercial availability of personal genome testing before it has attained sufficient levels of clinical validity and utility, may undermine public trust in genetics medicine and research [13][66]. It is argued that present-day genetic profiles are of such limited clinical validity that consumers will be disillusioned with their purchase, which could deprive genetics research of its chances to flourish. Changing interpretations may pose further threats to the public perception of clinical utility of personal genetic information [67].

Although personal genome testing implies its own potential societal risks, there is at least one such risk that has always been paramount to the ELSI-debate on clinical genetic testing, but that lacks ground in the context of genetic profiling for multifactorial diseases: the risk of discrimination or stigmatization. Genetic profiles of limited clinical validity will not be of interest to insurance companies or employers due to their limited utility for the purposes of risk stratification [68]. Indeed, genetics professionals today generally consider the risk of genetic discrimination to be very low [69].^12 ^In spite of widespread concern among ELSI-researchers, we therefore think that the fears of discrimination and stigmatization are not justified in the context of present-day personal genome testing.^13 ^We agree, however, that the risks of discrimination and stigmatization will again be of relevance to the ELSI-debate if personal genome testing gains sufficient clinical validity and becomes capable of reliably discriminating between individuals with high and low risks of developing multifactorial diseases.

### 3. Clinical validity: Regulatory issues

A third main ELSI associated with personal genome testing for multifactorial diseases is the legal and societal issue of regulation. The regulatory issue entails the ethical question whether it is morally justifiable to offer genetic tests of limited clinical validity and utility to the public, and if so, on what conditions. Within the regulatory debate, there are roughly two perspectives: the first emphasizes the value of protection and the second that of autonomy and consumer choice. From within the first perspective, there have been calls for enhanced government oversight and regulation [70-72], whereas the second perspective prioritizes respect for consumer liberty, to be complemented with governmental efforts to provide reliable information and to promote self-regulation of the market [73,74].

Within the first perspective, there are two separate and sometimes conflated positions: the position that genetic tests of unclear informational significance ought not to be offered direct-to-consumer, and the position that genetic testing in general must not be made available commercially (see Table 2). The first position presupposes that personal genome tests are of inferior clinical validity, and that they cannot be said to yield medical *information *at all. Personal genome tests are considered to be flawed as medical tests, or even as informational products [11], and thus, they ought not be put onto the market [13]. Contrarily, the second position presupposes that part of the information offered by direct-to-consumer personal genome testing companies may indeed be or become of clinical significance [25][75]. Analogous to clinical genetic testing, clinically valid direct-to-consumer personal genome testing is not without risk, it is claimed (see also section III,2). Therefore, personal genome testing ought not to be made available commercially, outside of the clinic. Prior consultation of a physician or a genetic counselor is or should be mandated in all genetic testing, in order to ensure adequate patient protection.

**Table 2:** Four positions on clinical validity and regulatory requirements

		Does personal genome testing have clinical validity?	
		**Yes (or some)**	**No (or not sufficiently)**

**Does personal genome testing require regulation?**	**Yes**	Because of potentially adverse health impact, and psychological and societal risks, personal genome testing ought to be made available only under medical supervision	Because of the risks of over-interpretation and subsequent health risks, personal genome testing ought not to be allowed on the market

	**No**	The risks are only minor, whereas access to (potentially or partly) useful genetic information is important and ought not to be hindered by regulatory restrictions	Personal genome testing is to be considered an informational or recreational product: consumer information is sufficient to regulate the market and to protect consumers from any risks

Within the second perspective, it can be maintained either that the risks of current direct-to-consumer personal genome testing of low clinical validity are not sufficiently serious to justify any infliction upon consumer autonomy and liberty of choice, or that the benefits of testing outweigh the risks (see Table 2). Either way, the second perspective states that patients or consumers ought to be allowed to make their own choices on the health care market, and that the availability of personal genome tests ought not to be restricted through government intervention. This means that even if there are psychological or health risks involved in personal genome testing for multifactorial diseases, competent consumers ought to be allowed, on the basis of adequate information, to make autonomous decisions regarding whether or not to undergo such testing.

We endorse the more liberal position within the regulatory debate, because we believe that the right to liberty of choice, where possible, must be respected in consenting adults. As discussed above, given the complexity and the quantity of the information, it will not always be easy for consumers to make rational and well-considered decisions with regard to the purchase of direct-to-consumer personal genome testing. We therefore believe that there are limits to the liberal position: providers may be required to make an extra effort to help their customers overcome the information problem.

### 4. Clinical utility: A personal approach to utility

Some groups of consumers appear to be attracted to personal genome testing for multifactorial diseases: some of the first empirical studies suggest that consumer interest is rather high and growing [20][76]. Thus, it seems that personal genome tests as *consumer products *have some sort of value. Over the last few years, the concept of clinical utility has been widened in order to account for that value. Notions of personal utility have been explored [35], and suggested in support of liberal attitudes towards direct-to-consumer personal genome testing [77].

The notion of personal utility is not unequivocal: it refers to different kinds of values, some more weighty than others. Whereas personal utility can refer to values such as a desire for certainty, an opportunity to prepare for the future, or possibilities for reproductive decision-making (see section I,4,1), there are also more 'frivolous' interpretations that align with the marketing rhetoric used by present-day personal genome testing companies. Apart from medical information, these companies offer genetic information about ancestry and other non-medical phenotypic traits, such as ear wax type, musculature type, eye colour or alcohol flush reaction. Personal genome testing services have been labelled 'recreational genomics' [16][78], and have been compared with astrology [78]. Not only are personal genetic tests marketed as a form of entertainment or even as a hobby [79], they are also presented as "a ticket to some sort of insight that amuses, edifies or helps one find one's place in society" [80]. On company websites, clients report having found out 'who they really are.'^14 ^Some companies stimulate consumers to share and compare their genetic make-up and to form online social networks around traits or medical conditions.

Critics have warned against the emphasis on the recreational value of personal genome testing: genetic tests cannot and must not be said to be purely (or even primarily) recreational when in fact they inform on (among other things) risks for serious medical conditions [81]. They have also questioned the capacities of consumers to assess personal utility. Consumers who believe that information obtained from personal genome testing is useful for them might have poor understanding and false expectations of the significance and the utility of that information [82]. Consumers may stop to perceive personal utility after having been informed thoroughly on the benefits and risks of non-targeted forms of genetic profiling. Studies have found that many people indeed tend to lose interest in genetic testing after having been informed about the limitations thereof [83-85].^15 ^Thus, the notion of personal utility of such tests could be questioned, as it may be based not so much upon considered valuations of consumers, but rather upon misconceptions that could partly be rebutted through the provision of information. In the absence of any clinical validity, we think that personal approaches to clinical utility, especially in the context of testing for disease risks, are unjustified.

On the other hand, in the presence of sufficient, reasonable or increasing levels of clinical validity, we believe that a personal approach toward utility may indeed be sensible: consumers may wish to decide for themselves whether informative non-targeted genetic profiling is valuable for them, and in what way. The high standards of clinical utility that are used within public health care evaluations need not be identical to the standards applicable to individual consumer valuations of personal utility. For example, consumers may find personal utility in knowing their genetic risk for Alzheimer's disease, despite the absence of preventive options. This issue deserves further elaboration, which is beyond the scope of the present article.

## Summary

For a well-informed and meaningful discourse on the ethical, legal and societal issues (ELSI) of present-day personal genome testing for multifactorial diseases, it is important to clarify the relevant test characteristics of personal genome tests. Test characteristics that are most essential to the current ELSI-debate are the following: non-targeted testing, high analytical validity, limited clinical validity, debatable clinical or personal utility, and the quantity, complexity and fluidity of the generated personal genetic risk information.

Non-targeted personal genome testing yields a vast amount of information that is complex and probabilistic, sometimes for a dozen to over a hundred multifactorial diseases simultaneously. Further, test outcomes may change over time as providers include additional genetic variants in their algorithms. Quantity, complexity, and fluidity of genetic information together pose urgent problems with regard to the provision of information and informed consent. Providers of personal genome testing are facing these informational problems at several moments within the testing process: pre-test informed consent, post-test delivery of test results, and post-test dealing with future (incidental) findings and changing interpretations. There is a pressing need for well thought-out models for valid informed consent and information provision in the context of a lot of complex and fluid information in non-targeted personal genome testing.

Since personal genome testing is increasingly based on highly accurate and reliable genome-wide SNP-scanning technology and performed in high-quality laboratories, the test characteristic analytical validity has moved away from the centre of ELSI-discussions. Current debates are focused rather on the clinical validity and utility of genetic profiles for multifactorial diseases, which vary strongly, but are likely to increase given time. Awareness of the currently limited clinical validity is at the basis of both conservative and liberal stances within the regulatory discourse: it is used either as an argument in favor of stricter regulation, or as an argument against it.

The notion of clinical utility is challenged by personal approaches towards the significance and usefulness of genetic information. It is far from impossible that consumers continue to attribute personal utility to genetic information and pursue the acquisition of their genomic data even after having been informed about the current clinical limitations of genetic profiling for multifactorial diseases. Standards of clinical utility that are used for public health evaluations, however, need not be identical to those used for individual valuations of utility.

As a consequence of their limited clinical validity, present-day personal genome tests for multifactorial diseases have a much lower potential for adverse psychological effects than do clinical genetic tests for monogenic diseases. Neither do they imply as many health risks, or societal risks, such as discrimination, stigmatization and misuse of genetic information by insurance companies or employers. This holds true only on the condition that the general public as well as other stake-holding parties are sufficiently informed to understand the limitations to the clinical validity and utility of genetic profiling for multifactorial diseases, and are willing to act accordingly. In the future, as genetic profiles will attain more discriminative ability, both traditional psychological risks and concurrent health and societal risks will again be of concern to the discourse on ELSI.

The applicability and the relevance of ELSI-issues to the discourse on personal genome testing will fluctuate with the analytical and clinical validity of genetic profiles, with their clinical utility and with their being targeted or non-targeted. Thus, consideration of test characteristics is indispensible to any valuable ELSI-debate on personal genome testing for multifactorial diseases.

## Competing interests

The authors declare that they have no competing interests.

## Authors' contributions

All authors have contributed substantially to the conception and design of the manuscript. EB has drafted the manuscript. Both MS and AJ have critically revised it. All authors have read and approved the final manuscript.

## Notes

1. To the knowledge of the authors, the company Knome (pronounced as 'know me') is the only direct-to-consumer provider of whole-genome sequencing that offers (among other tests) risk profiling for multifactorial diseases. See: http://www.knome.com/ (Accessed June 13^th^, 2011)

2. See for example Navigenics at http://www.navigenics.com or Pathway Genomics at http://www.pathway.com (Accessed June 13^th^, 2011)

3. See http://www.23andme.com (Accessed June 13^th^, 2011). Currently, the company 23andme offers risk profiles for 195 diseases and other phenotypic traits, but the number of traits tested for increases monthly. The company also provides ancestry and carrier status information. While most other direct-to-consumer personal genome testing companies are currently offering scans for no more than a few dozen diseases, they are likely to expand their services in the future rather than restrict them.

4. Most US-based companies collaborate with CLIA-certified laboratories, see for example http://www.knome.com (Accessed June 13^th^, 2011). With the Clinical Laboratory Improvement Amendment (CLIA) of 1988, the US government has set quality standards for all laboratory tests, ensuring their accuracy, reliability, and timeliness.

5. The company 23andme states that it makes use of a chip which demonstrates over 99.9% reproducibility: "This means that if [the laboratory] ran the same DNA a second time on a new chip, more than 99.9% of the data would be the same compared to data from the first run" https://www.23andme.com/you/faqwin/dataaccuracy/. (Accessed June 13^th^, 2011)

6. There are other diseases that are more promising for predictive genetic profiling: SNPs have been found to be associated with almost 3-fold risks for age-related macular degeneration (AMD). Genetic profiles for AMD have already been made available online (https://www.23andme.com/health/Age-related-Macular-Degeneration/techreport/, http://www.arcticdx.com/ (Accessed June 13^th^, 2011)). Relatively strong predictive abilities such as those in genetic profiling for AMD are far from typical for common multifactorial diseases.

7. The fluidity of test results depends in part on the clinical validity of the existing profile: the higher that validity, the less likely it will change with the advancement of genetics research and the inclusion of additional markers.

8. The extent to which the problem of informed consent is present, however, will depend on various aspects, as it will be easier to effectively provide information, for instance, if the amount of diseases tested for is smaller.

9. As these statements have been written before current personal genome testing companies started to offer genome-wide non-targeted genetic profiling, it is not self-evident that the terms "all genetic testing" in these statements also include present-day commercial services.

10. See for example 23andme at http://www.23andme.com or Navigenics at http://www.navigenics.com (Accessed June 13^th^, 2011).

11. See https://www.23andme.com/health/Colorectal-Cancer/ (Accessed June 13^th^, 2011)

12. Respondents were US cancer genetics professionals involved in highly predictive genetic testing for familial cancer syndromes. Respondents may be assumed to consider the risk of genetic discrimination to be lower in case of genetic profiling in the context of multifactorial diseases.

13. It should be noted that misuse and abuse of genetic risk information by employers or insurance companies cannot be excluded completely, for employers and insurance companies are susceptible to misinterpretation of genetic test results, and to overstatement of their significance.

14. See for example Pathway Genomics at http://www.pathway.com (Accessed June 13^th^, 2011).

15. These studies have been conducted in the context of genetic testing for highly predictive single-gene makers, such as BRCA1 and BRCA2. Women had been informed, for example, that genetic test results are often inconclusive and that they are of unclear significance in the absence of a family history of breast cancer [68,70]. As the results provided by most genetic profiling services are much more uncertain, one would expect the effect of disappointment found in these studies to be increased for personal genome testing.

## Pre-publication history

The pre-publication history for this paper can be accessed here:

http://www.biomedcentral.com/1472-6939/12/11/prepub
